# Association of insomnia and daytime napping with metabolic syndrome and its components in a Korean population: an analysis of data from the Korean Genome and Epidemiology Study

**DOI:** 10.4178/epih.e2024031

**Published:** 2024-02-20

**Authors:** Da-Been Lee, Dae-Wui Yoon, Inkyung Baik

**Affiliations:** 1 Department of Health and Environmental Science, Korea University, Seoul, Korea; 2 Sleep Medicine Institute, Jungwon University, Goesan, Korea; 3 Department of Biomedical Laboratory Science, Jungwon University, Goesan, Korea; 4 Department of Foods and Nutrition, Kookmin University College of Natural Sciences, Seoul, Korea

**Keywords:** Sleep, Metabolic syndrome, Prevalence

## Abstract

**OBJECTIVES:**

We investigated the association between metabolic syndrome (MetS) and the coexistence of insomnia and daytime napping, because limited data have been reported regarding this association.

**METHODS:**

The study population was 8,440 participants aged 40-65 years, who were from the Korean Genome and Epidemiology Study. Self-reported information on insomnia symptoms and nap duration was used to define exposure variables. Data on waist circumference (WC), blood pressure (BP), and fasting blood glucose (FBG), triglyceride (TG), and high-density lipoprotein cholesterol levels in blood were used to define MetS. Multivariate logistic regression analysis was performed to obtain odds ratio (OR) and 95% confidence interval (CI).

**RESULTS:**

In multivariate logistic regression analysis, the coexistence of insomnia and napping was not significantly associated with MetS. However, the insomnia and non-napping group showed higher ORs of high TG (OR, 1.19; 95% CI, 1.02 to 1.39) and high BP (OR, 1.28; 95% CI, 1.10 to 1.49) than the non-insomnia and non-napping group. The combination of non-insomnia and napping and that of insomnia and napping showed higher ORs of high TG (OR, 1.13; 95% CI, 1.00 to 1.29) and high FBG (OR, 1.59; 95% CI, 1.14 to 2.21), respectively. In analyses of insomnia symptoms, only the combination of difficulty in maintaining sleep (DMS) and non-napping showed a higher OR for MetS (OR, 1.25; 95% CI, 1.03 to 1.52) than the non-DMS and non-napping group.

**CONCLUSIONS:**

Individuals with insomnia, particularly those who do not take naps, were disproportionately likely to have MetS components, especially TG or BP. Information on these variables may help predict individuals’ vulnerability to specific MetS components.

## GRAPHICAL ABSTRACT

[Fig f1-epih-46-e2024031]

## Key Message

• The significant association of insomnia with MetS components varies with the presence or absence of napping.

• Individuals with insomnia who do not take naps were likely to have MetS components, especially TG or BP.

## INTRODUCTION

Metabolic syndrome (MetS) is a set of 5 conditions that increase the likelihood of developing cardiovascular and metabolic diseases. Individuals with MetS face a two-fold increase in the risk of cardiovascular disease (CVD) and are 5 times more likely to develop diabetes mellitus than those without MetS [1-3].

Overweight and obesity, lifestyle habits such as smoking, drinking, and shift work, genetic factors, sex, and medical conditions are recognized as risk factors for MetS. Recent research indicates that both the quantity and quality of sleep are linked to MetS. A population-based study utilizing data from the 2013-2014 National Health and Nutrition Examination Survey (NHANES) found a U-shaped relationship between sleep duration and the risk of MetS [4]. Numerous cross-sectional and longitudinal studies have identified a significant relationship between insomnia and MetS. One longitudinal study investigating the link between insomnia and MetS, along with its components, revealed that transitioning from restful sleep to insomnia was associated with an increased risk of developing MetS and hypertriglyceridemia [5]. Additionally, several cross-sectional studies in various ethnic groups have demonstrated independent associations between insomnia or specific symptoms of insomnia and MetS [6-8].

In addition to complaints of nighttime sleep disturbances, daytime napping has also been linked to MetS. Studies have demonstrated that habitual napping is independently associated with an increased prevalence [9] and incidence [10] of MetS and its components.

While research on the effects of napping on MetS has consistently yielded negative results, napping has been shown to have a positive impact on counteracting the bodily dysfunctions caused by sleep loss or sleep complaints. Non-restorative sleep (NRS) has been associated with an increased risk of developing hypertension (HTN) [11]. Xu et al. [12] reported that napping could reverse the cognitive impairments caused by sleep deprivation, and another study indicated that abstaining from napping significantly reduced episodic memory [13]. These findings suggest that in cases of sleep issues such as sleep deprivation or insomnia, recuperative sleep obtained through napping can have beneficial effects on the body.

Although insomnia and napping have each been individually associated with MetS and its components, to our knowledge, there has been no report on the association between the co-occurrence of insomnia and daytime napping with MetS. Therefore, this study evaluated the associations between the combination of insomnia and napping with MetS and its components in Korean adults.

## MATERIALS AND METHODS

### Study population

The current study utilized data from the Ansan and Ansung cohort study, which were obtained from the Korea Disease Control and Prevention Agency. A detailed description of these cohort studies is available in previous reports [14,15]. Briefly, the cohort studies commenced in 2001 as part of the Korean Genome and Epidemiology Study. Approximately 10,000 Korean male and female adults, aged 40 years to 69 years and residing in Ansan and Ansung, were enrolled as cohort members. They were invited to undergo health examinations on-site from June 2001 to January 2003. In total, 9,996 members completed the examination, which included anthropometric and clinical assessments, blood collection for biochemical and genetic assays, and a questionnaire-based interview. The questionnaire covered socio-demographics, medical history and health conditions, and lifestyle factors, including sleep status.

### Definitions of daytime napping, insomnia, and metabolic syndrome

In the questionnaire-based interview, participants were asked to detail their sleep patterns, including nighttime sleep duration, daytime napping frequency and duration, and the presence of insomnia symptoms. If participants indicated that they took daytime naps, they were further queried about the average minutes spent napping each day. Based on their responses, napping was categorized as taking a nap for more than 1 hour, while non-napping was defined as either not napping at all or taking a short nap of less than 1 hour. This categorization aligns with definitions used in prior studies [9,16]. The information on insomnia symptoms was available in the questionnaire and used to define insomnia. Participants responded to the following questions: (1) “Do you have difficulty falling asleep at night?” (difficulty initiating sleep, DIS), (2) “Do you wake up during the night and have difficulty falling back asleep?” (difficulty maintaining sleep, DMS), (3) “Do you wake up too early in the morning?” (early morning awakenings, EMA), and (4) “Do you feel refreshed when you woke up in the morning?” (non-restorative sleep, NRS). They were instructed to answer “yes” or “no” to these questions. For affirmative responses, they were asked to specify the frequency of their experiences, with options such as very rarely, sometimes, often, and almost every day (1= never, 2= 1-2, 3= 3-4, and 4= ≥ 5 times/wk). Individuals reporting any of these 4 symptoms with a frequency of more than 3-4 times/wk (score ≥ 3 on any item) were classified as having insomnia symptoms, irrespective of their use of sleep medication. Groups with insomnia were then formed based on the frequency of reported insomnia symptoms at each time point.

MetS was defined as the presence of ≥ 3 of the following 5 criteria, according to the National Cholesterol Education Program Adult Treatment Panel III [17]: (1) abdominal obesity, characterized by a waist circumference of ≥ 90 cm for male and ≥ 80 cm for female; (2) hypertriglyceridemia, with serum triglyceride (TG) levels of ≥ 150 mg/dL; (3) low high-density lipoprotein (HDL)- cholesterol, defined as < 40 mg/dL for male and < 50 mg/dL for female; (4) HTN, indicated by a systolic/diastolic pressure of ≥ 130/85 mmHg or the use of antihypertensive medications; (5) high fasting blood glucose (FBG), with levels of ≥ 100 mg/dL or the use of antidiabetic medications.

### Anthropometric and biochemical measurements

All participants underwent anthropometric measurements. Weights and heights were recorded to the nearest 0.1 kg and 0.1 cm, respectively. Subsequently, body mass index (BMI) was calculated by dividing the weight (kg) by the square of the height (m^2^). Blood pressure (BP) was measured non-invasively while participants were seated, using a mercury sphygmomanometer by trained personnel. Physical activity levels were quantified in metabolic equivalent (MET)-hr/day. Blood samples were collected from participants after an overnight fast. Serum levels of blood glucose, TG, and HDL-cholesterol were measured using an ADVIA 1650 autoanalyzer (Siemens, Tarrytown, NY, USA) at a commercial laboratory (Seoul Clinical Laboratories, Seoul, Korea).

### Statistical analysis

Data are expressed as mean± standard deviation. One-way analysis of variance for continuous variables and the chi-square test for categorical variables were used. Univariate and multivariate logistic regression analyses were conducted to calculate odds ratios (ORs) and 95% confidence intervals (CIs) for the associations of MetS and MetS components with napping, insomnia, and insomnia symptoms. Age (continuous), sex (male or female), BMI (continuous), education level (high school or college), smoking status (non-smoker or current smoker), alcohol consumption status (past drinker or current drinker), physical activity (MET-hr/day; continuous), and presence of obstructive sleep apnea (OSA; no or yes) were included as covariates in the multivariate logistic regression analysis. All statistical analyses were performed using SAS version 9.4 (SAS Institute Inc., Cary, NC, USA), and a p-value < 0.05 was considered statistically significant.

### Ethics statement

All participants provided written informed consent at 2 study sites, Korea University Ansan Hospital and Ajou University Medical Center. The current study received approval from the Institutional Review Board of Jungwon University (1044297-HR-202306-002-01).

## RESULTS

### Characteristics of the study population

Table 1 presents the general characteristics of the study population. Out of the 8,440 participants with complete data, 2,490 (29.5%) were diagnosed with MetS. Those with MetS tended to be older, have a higher body weight, and were more likely to be male, current smokers, and current drinkers than participants without MetS. Among the participants, 20.3% reported regular daily napping, while 64.9% experienced insomnia. The frequency of napping did not significantly differ between those with and without MetS. However, the prevalence of insomnia was significantly higher in participants with MetS. Notably, the symptoms of EMA and NRS were more common among those with MetS. Significant differences were also observed in sleep duration and the number of OSA events between participants with and without MetS.

Table 2 shows the characteristics of participants with regard to napping and insomnia. Education level and sleep duration were the only variables that significantly differed between the napping and non-napping groups. Participants with insomnia were generally older, heavier, and more physically active than those without insomnia. A higher proportion of female than male was found among participants with insomnia. Those with insomnia also had a higher prevalence of MetS, as well as significantly greater proportions of abdominal obesity, high TG, and high BP compared to those without insomnia. Additionally, there was significant variation in the number of participants when categorized by sleep duration.

### Associations of metabolic syndrome with the coexistence of insomnia and napping

Table 3 shows the associations of MetS with the coexistence of insomnia and napping. Compared to the non-insomnia and non-napping group, the coexistence of insomnia and non-napping had higher ORs for MetS in the crude analysis, but this was no longer significant after controlling for confounding variables.

### Associations of metabolic syndrome components with the coexistence of insomnia and napping

Next, we conducted a multivariate logistic regression analysis to examine the associations of individual MetS components with the coexistence of insomnia and napping (Table 4). MetS components were significantly associated with different combinations of napping and insomnia. The coexistence of insomnia and napping was significantly associated with higher TG levels compared to the coexistence of non-insomnia and napping (OR, 1.13; 95% CI, 1.00 to 1.29; p= 0.047) and the coexistence of insomnia and non-napping (OR, 1.19; 95% CI, 1.02 to 1.39; p= 0.026). Significant associations were found between the coexistence of insomnia and non-napping and high BP (OR, 1.28; 95% CI, 1.10 to 1.49; p= 0.002). In addition, the coexistence of insomnia and napping was associated with a higher likelihood of elevated FBG (OR, 1.59; 95% CI, 1.14 to 2.21; p= 0.006), compared to the non-insomnia and non-napping. No significant interaction was observed between insomnia and napping in terms of associations with MetS components, although borderline significance was found for high BP.

### Associations of metabolic syndrome with the coexistence of insomnia symptoms and napping

As shown in Table 5, the combination of DMS and non-napping was associated with a higher OR (OR, 1.28; 95% CI, 1.07 to 1.54; p = 0.009) for MetS compared to the non-DMS and nonnapping group in the crude model. The OR for MetS decreased slightly, but remained significant after adjusting for confounders (OR, 1.25; 95% CI, 1.03 to 1.52; p = 0.023). The coexistence of EMA and non-napping, NRS and non-napping, and NRS and napping had significant positive associations with MetS in the crude analysis, but these associations disappeared after adjusting for confounders.

## DISCUSSION

This study investigated the associations of the combination of insomnia and napping with MetS or its components in a large, population-based study. While no group defined by the combination of insomnia and napping was associated with MetS as a whole, individuals with insomnia who did not nap exhibited a greater likelihood of having certain MetS components, including high TG and high BP. Specifically, DMS was linked to MetS, particularly in those who did not nap.

Contradictory results have been reported regarding the effects of daytime napping on CVD. A Greek cohort study [18] reported that daytime napping was linked to a 34% decrease in the risk of death from coronary heart disease. Similarly, another populationbased, prospective cohort study reported a significantly lower risk of incident cardiovascular events for individuals who napped once or twice per week compared to those who did not nap [19]. In contrast, other cohort studies [20-22] and a case-control study [23] have shown an increased risk for heart disease or CVD mortality. A dose-response meta-analysis of 20 cohort studies revealed that longer napping durations (> 60 min/day) were associated with a heightened risk of CVD. This suggests that both the frequency and duration of napping may influence the relationship between daytime napping and CVD risk [24].

Unlike the conflicting relationships between napping and CVD, relatively consistent findings have been reported regarding the association between napping and metabolic disease. A previous meta-analysis demonstrated a J-shaped association between napping and the risk of metabolism-related diseases [25], and this relationship appears to vary by napping duration, age, sex, and specific metabolic conditions. In a Chinese population-based study, daytime napping for more than 1 hour was independently associated with a higher prevalence of diabetes compared to the nonnapping group, but daytime napping for a half hour or less was associated with a lower prevalence of fatty liver and dyslipidemia only in female aged less than 50 years old [26]. We also observed that individuals without insomnia who napped were more likely to have elevated TG levels. The mechanism underlying these associations between napping and metabolic diseases is still unclear, but possible mediators include evening cortisol elevation [27], sympathetic nervous system activation after napping [28], and disrupted circadian rhythms [29]. Additionally, since napping is directly linked to longer durations spent in bed, it may lead to decreased energy expenditure and an increase in fat deposition, which could also contribute to higher TG levels [30].

Poor sleep quality, or specific types of sleep complaints, can differentially affect MetS and its components. A recent meta-analysis has shown that poor sleep quality is linked to several components of MetS, including elevated blood pressure, an abnormal lipid profile, and impaired glycemic control [31]. In a cross-sectional study investigating the relationship between insomnia and MetS, insomnia was significantly associated only with low HDL-cholesterol and high serum TG, indicating that dyslipidemia is a primary MetS component influenced by insomnia [8]. Certain symptoms of insomnia may predict the onset of MetS. For instance, in a community-based, 3-year follow-up study, DIS and NRS were significant predictors of MetS development [32]. Another cross-sectional study examining the link between individual insomnia symptoms and MetS found that, of the symptoms, only DMS was significantly associated with MetS, which aligns with our findings [33]. Collectively, both cross-sectional and longitudinal studies have reported a significant association between insomnia and MetS, with an increased risk of developing MetS in individuals with insomnia [6,34,35], and significant relationships have been found between specific insomnia symptoms and components of MetS [5,33,35].

Previous studies have demonstrated a positive association between napping and abdominal obesity [36,37]. However, our findings did not reveal this relationship, which may be attributed to variations in how napping and obesity are defined, as well as the differences in the confounders that were adjusted for in our analysis. Our findings indicate that a single exposure to either napping or insomnia is associated with elevated TG, while the concurrent presence of both insomnia and napping does not show this association. This observation aligns with prior research that has established a connection between napping or insomnia and an increased risk of cardiovascular and metabolic conditions [38,39].

The present study also showed that individuals with insomnia, especially those who do not take naps, are more likely to have high BP or elevated TG. Sleep deprivation or poor sleep quality in those with insomnia triggers biological responses, including activation of the hypothalamic-pituitary-adrenal (HPA) axis and the sympathetic nervous system (SNS), as well as inflammation [40-42], all of which can lead to increased BP. Furthermore, a lack of sleep has been linked to changes in appetite-regulating hormones [43] and a heightened desire for carbohydrate-rich foods [44]. Therefore, napping may help individuals with insomnia counteract these biological responses by compensating for lost sleep or poor sleep quality.

However, we found adverse results for the coexistence of insomnia and napping in association with high FBG. These results are somewhat unexpected, given that napping can reduce the activity of the HPA axis and SNS, which are well-known blood glucose elevators. The timing and frequency of napping, the type of insomnia symptoms, and the presence of uncontrolled confounding variables may influence the synergistic association between the combination of insomnia and napping and high FBG.

Several limitations should be considered when interpreting the results of this study. First, the study’s cross-sectional design precludes the inference of a causal relationship for the observed associations. Second, the exposure variables, insomnia and daytime napping, were defined based on self-reported questionnaires, which may be less accurate than data obtained from a doctor’s diagnosis or a sleep recording device. Third, given that the components of MetS are interrelated, the results from multiple testing with correlated outcomes require cautious interpretation, even though each component was analyzed in a separate model.

In summary, this population-based cross-sectional study showed that individuals with insomnia, particularly those who do not nap, are likely to have MetS components, such as TG or BP. Further studies are needed to provide causal inferences regarding the association of coexisting insomnia and napping with MetS and its components.

## Figures and Tables

**Figure f1-epih-46-e2024031:**
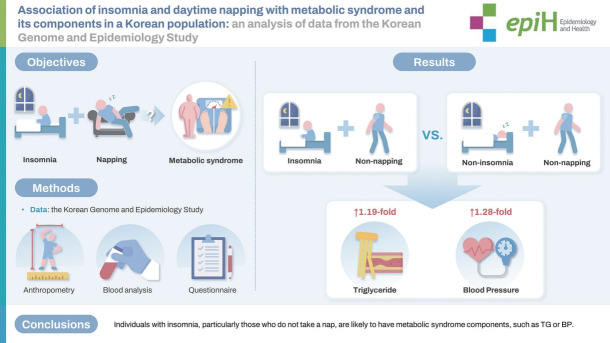


**Table 1.** Characteristics of the study population according to the presence of MetS

Characteristics	Total (n=8,440)	MetS		p-value
		Yes (n=2,490)	No (n=5,950)	
Age (yr)	50.46±7.72	52.17±7.75	49.74±7.60	<0.001
Sex				<0.001
Female	4,388 (52.0)	953 (38.4)	3,435 (57.7)	
Male	4,052 (48.0)	1,537 (61.7)	2,515 (42.3)	
BMI (kg/m^2^)	24.66±3.09	26.33±2.82	23.95±2.92	<0.001
Education				0.061
≤High school	7,225 (85.6)	2,104 (84.5)	5,121 (86.1)	
≥College	1,215 (14.4)	386 (15.5)	829 (13.9)	
Smoking status				<0.001
Non-smoker	6,278 (74.4)	1,704 (68.4)	4,574 (76.9)	
Current smoker	2,162 (25.6)	786 (31.6)	1,376 (23.1)	
Alcohol consumption				<0.001
Past drinker	4,534 (53.7)	1,213 (48.7)	3,321 (55.8)	
Current drinker	3,906 (46.3)	1,277 (51.3)	2,629 (44.2)	
Physical activity (MET-hr/day)	30.65±15.20	30.72±15.48	30.62±15.1	0.796
Napping				0.159
No	6,724 (79.7)	1,960 (78.7)	4,764 (80.1)	
Yes	1,716 (20.3)	530 (21.3)	1,186 (19.9)	
Insomnia				0.001
No	2,966 (35.1)	795 (31.9)	2,171 (36.5)	
Yes	5,474 (64.9)	1,695 (68.1)	3,779 (63.5)	
Insomnia symptoms				
DIS	514 (6.1)	157 (6.3)	357 (6.0)	0.887
DMS	674 (8.0)	226 (9.1)	448 (7.5)	0.058
EMA	682 (8.1)	230 (9.2)	452 (7.6)	0.034
NRS	4,795 (56.8)	1,475 (59.2)	3,320 (55.8)	0.004
MetS components				
Abdominal obesity	3,725 (44.1)	2,026 (81.4)	1,699 (28.5)	<0.001
High triglyceride	3,105 (36.8)	2,055 (82.5)	1,050 (17.6)	<0.001
Low HDL-cholesterol	3,162 (37.5)	1,594 (64.0)	1,568 (26.3)	<0.001
High blood pressure	3,405 (40.3)	1,859 (74.7)	1,546 (26.0)	<0.001
High blood glucose	1,486 (17.6)	1,076 (43.2)	410 (6.9)	<0.001
Sleep duration (hr)				0.004
<5	396 (4.7)	124 (5.0)	272 (4.6)	
5-6	969 (11.5)	271 (10.9)	698 (11.7)	
6-7	2,224 (26.3)	607 (24.4)	1,617 (27.18)	
7-8	2,497 (29.6)	730 (29.3)	1,767 (29.7)	
8-9	1,730 (20.5)	544 (21.8)	1,186 (19.9)	
>9	624 (7.4)	214 (8.6)	410 (6.9)	
OSA				<0.001
No	7,931 (94.0)	2,297 (92.2)	5,634 (94.7)	
Yes	509 (6.0)	193 (7.7)	316 (5.3)	

Values are presented as mean±standard deviation or number (%). BMI, body mass index; MET, metabolic equivalent; DMS, difficulty in maintaining sleep; EMA, early morning awakening; NRS, non-restorative sleep; MetS, metabolic syndrome; HDL, high-density lipoprotein; OSA, obstructive sleep apnea.

**Table 2.** Characteristics of the study population according to the napping and insomnia

Characteristics	Napping		p-value	Insomnia		p-value
	Yes (n=1,716)	No (n=6,724)		Yes (n=5,474)	No (n=2,966)	
Age (yr)	50.42±7.69	50.47±7.73	0.821	51.30±7.80	48.91±7.33	<0.001
Sex			0.162		<0.001	
Female	918 (53.5)	3,470 (51.6)		2,732 (49.9)	1,320 (44.5)	
Male	798 (46.5)	3,254 (48.4)		2,742 (50.1)	1,646 (55.5)	
BMI (kg/m^2^)	24.69±3.11	24.65±3.08	0.628	24.74±3.08	24.51±3.10	0.001
Education			<0.001		0.744	
≤High school	1,531 (89.2)	5,694 (84.7)		4,691 (85.7)	2,534 (85.4)	
≥College	185 (10.8)	1,030 (15.3)		783 (14.3)	432 (14.6)	
Smoking status			0.696			0.509
Non-smoker	1,278 (74.5)	5,000 (74.4)		4,087 (74.7)	2,191 (73.9)	
Current smoker	438 (25.5)	1,724 (25.6)		1,387 (25.3)	775 (26.1)	
Alcohol consumption			0.429			0.195
Past drinker	940 (54.8)	3,594 (53.4)		2,962 (54.1)	1,572 (53.0)	
Current drinker	776 (45.2)	3,130 (46.5)		2,512 (45.9)	1,394 (47.0)	
Physical activity (MET-hr/day)	30.10±14.78	30.79±15.30	0.097	31.05±15.39	29.91±14.82	0.001
Napping						0.141
No				4,387 (80.1)	2,337 (78.8)	
Yes				1,087 (19.9)	629 (21.2)	
Insomnia			0.141			
No	629 (36.7)	2,337 (34.8)				
Yes	1,087 (63.3)	4,387 (65.2)				
Insomnia symptoms						
DIS	93 (5.4)	421 (6.3)	0.247	514 (9.4)	0 (0.0)	<0.001
DMS	128 (7.5)	546 (8.1)	0.083	674 (12.3)	0 (0.0)	<0.001
EMA	117 (6.8)	565 (8.4)	0.170	682 (12.5)	0 (0.0)	<0.001
NRS	960 (55.9)	3,835 (57.0)	0.416	4,795 (87.6)	4,343 (51.5)	<0.001
MetS			0.159			<0.001
No	1,186 (69.1)	4,764 (70.8)		3,779 (69.0)	2,171 (73.2)	
Yes	530 (30.9)	1,960 (29.1)		1,695 (31.0)	795 (26.8)	
MetS components						
Abdominal obesity	774 (45.1)	2,951 (43.9)	0.365	2,530 (46.2)	1,195 (40.3)	<0.001
High triglyceride	657 (38.3)	2,448 (36.4)	0.150	2,075 (37.9)	1,030 (34.7)	0.004
Low HDL-cholesterol	671 (39.1)	2,491 (37.0)	0.116	2,049 (37.4)	1,113 (37.5)	0.932
High blood pressure	662 (38.6)	2,743 (40.8)	0.095	2,316 (42.3)	1,089 (36.7)	<0.001
High blood glucose	324 (18.9)	1,162 (17.3)	0.120	992 (18.1)	494 (16.7)	0.091
Sleep duration (hr)			<0.001			<0.001
<5	107 (6.2)	289 (4.3)		303 (5.5)	93 (3.1)	
5-6	211 (12.3)	758 (11.3)		598 (10.9)	371 (12.5)	
6-7	394 (23.0)	1,830 (27.2)		1,395 (25.5)	829 (27.9)	
7-8	473 (27.6)	2,024 (30.1)		1,618 (29.6)	879 (29.6)	
8-9	372 (21.7)	1,358 (20.2)		1,143 (20.9)	587 (19.8)	
>9	159 (9.3)	465 (6.9)		417 (7.6)	207 (7.0)	
OSA			0.334			0.693
No	1,604 (93.5)	6,327 (94.1)		5,148 (94.0)	2,783 (93.8)	
Yes	112 (6.5)	397 (5.9)		326 (6.0)	183 (6.2)	

Values are presented as mean±standard deviation or number (%). BMI, body mass index; MET, metabolic equivalent; DIS, difficulty in initiating sleep; DMS, difficulty in maintaining sleep; EMA, early morning awakening; NRS, non-restorative sleep; MetS, metabolic syndrome; HDL, high-density lipoprotein; OSA, obstructive sleep apnea.

**Table 3.** Association of the combination of insomnia and napping with MetS^1^

Exposures	MetS				
	Case/Non-case	Crude OR (95% CI)	p-value	Multivariate OR (95% CI)	p-value
Non-insomnia & non-napping	1,659/4,148	1.00 (reference)		1.00 (reference)	
Non-insomnia & napping	461/1,041	1.11 (0.98, 1.25)	0.106	1.11 (0.98, 1.27)	0.101
Insomnia & non-napping	301/616	1.22 (1.05, 1.42)	0.009	1.15 (0.98, 1.35)	0.081
Insomnia & napping	69/145	1.19 (0.89, 1.59)	0.244	1.11 (0.82, 1.51)	0.486

MetS, metabolic syndrome; OR, odds ratio; CI, confidence interval.

1 Data were adjusted for age, sex, body mass index, smoking status, alcohol consumption status, and the presence of obstructive sleep apnea.

**Table 4.** Association of the combination of insomnia and napping with metabolic syndrome components

Group	Non-insomnia & non-napping		Non-insomnia & napping		Insomnia & non-napping		Insomnia & napping		p-value for interaction
	Case/Non-case	Multivariate OR (95% CI)	Case/Non-case	Multivariate OR (95% CI)	Case/Non-case	Multivariate OR (95% CI)	Case/Non-case	Multivariate OR (95% CI)	
Abdominal obesity (n=3,725)^1^	2,572/3,235	1.00 (reference)	676/826	1.13 (0.99, 1.30)	379/538	1.04 (0.88, 1.23)	98/116	1.18 (0.85, 1.63)	0.992
High TG (n=3,105)^2^	2,080/3,727	1.00 (reference)	576/926	1.13 (1.00, 1.29)^*^	368/549	1.19 (1.02, 1.39)^*^	81/133	1.06 (0.78, 1.42)	0.163
Low HDL-c (n=3,162)^2^	2,113/3,694	1.00 (reference)	588/914	1.09 (0.96, 1.23)	378/539	1.08 (0.93, 1.26)	83/131	1.00 (0.74, 1.36)	0.367
High BP (n=3,405)^2^	2,287/3,520	1.00 (reference)	575/927	0.94 (0.83, 1.06)	456/461	1.28 (1.10, 1.49)^**^	87/127	0.87 (0.65, 1.18)	0.075
High FBG (n=1,486)^2^	986/4,821	1.00 (reference)	270/1,232	1.07 (0.92, 1.24)	176/741	1.12 (0.93, 1.35)	54/160	1.59 (1.14, 2.21)^**^	0.153

OR, odds ratio; CI, confidence interval; TG, triglyceride; HDL-c, high-density lipoprotein-cholesterol; BP, blood pressure; FBG, fasting blood glucose.

1 Data were adjusted for age, sex, smoking status, alcohol consumption status, and the presence of obstructive sleep apnea in case of abdominal obesity.

2 Data were adjusted for age, sex, body mass index, smoking status, alcohol consumption status, and the presence of obstructive sleep apnea in case of high TG, low HDL-c, high BP, and high FBG.

* p<0.05,

** p<0.01.

**Table 5.** Associations of combinations of insomnia symptoms and napping with MetS

Exposures	MetS				
	Case/non-case	Crude OR (95% CI)	p-value	Multivariate OR (95% CI)	p-value
Non-DIS & non-napping	1,830/4,473	1.00 (reference)		1.00 (reference)	
Non-DIS & napping	503/1,120	1.10 (0.98, 1.24)	0.123	1.10 (0.97, 1.25)	0.128
DIS & non-napping	130/291	1.09 (0.88, 1.35)	0.421	1.10 (0.89, 1.38)	0.381
DIS & napping	27/66	1.00 (0.64, 1.57)	1.000	1.06 (0.67, 1.69)	0.799
Non-DMS & non-napping	1,774/4,404	1.00 (reference)		1.00 (reference)	
Non- DMS & napping	490/1,098	1.11 (0.98, 1.25)	0.094	1.12 (0.99, 1.26)	0.086
DMS & non-napping	186/360	1.28 (1.07, 1.54)	0.009	1.25 (1.03, 1.52)	0.023
DMS & napping	40/88	1.13 (0.77, 1.65)	0.531	1.08 (0.73, 1.60)	0.692
Non-EMA & non-napping	1,773/4,386	1.00 (reference)		1.00 (reference)	
Non-EMA & napping	487/1,112	1.08 (0.96, 1.22)	0.191	1.09 (0.96, 1.23)	0.182
EMA & non-napping	187/378	1.22 (1.02, 1.47)	0.031	1.09 (0.90, 1.32)	0.394
EMA & napping	43/74	1.44 (0.98, 2.10)	0.061	1.24 (0.84, 1.85)	0.278
Non-NRS & non-napping	795/2,094	1.00 (reference)		1.00 (reference)	0.160
Non-NRS & napping	220/536	1.08 (0.91, 1.29)	0.388	1.14 (0.95, 1.37)	
NRS & non-napping	1,165/2,670	1.15 (1.03, 1.28)	0.011	1.03 (0.92, 1.15)	0.582
NRS & napping	310/650	1.26 (1.07, 1.47)	0.005	1.09 (0.92, 1.29)	0.306

Data were adjusted for age, sex, body mass index, smoking status, alcohol consumption status, and the presence of obstructive sleep apnea. MetS, metabolic syndrome; OR, odds ratio; CI, confidence interval; DIS, difficulty in initiating sleep; DMS, difficulty in maintaining sleep; EMA, early morning awakening; NRS, non-restorative sleep.
